# Characterization and Electrochemical Behaviour of Nanoscale Hydrotalcite-Like Compounds toward the Reduction of Nitrate

**DOI:** 10.3390/nano10101926

**Published:** 2020-09-27

**Authors:** Liang Li, Junhao Yang, Yafeng Yun, Shouxun Hu, Yuanxing Huang

**Affiliations:** School of Environment and Architecture, University of Shanghai for Science and Technology, Shanghai 200093, China; liliang@usst.edu.cn (L.L.); 183841881@st.usst.edu.cn (J.Y.); yunyafeng@shgfcz.com (Y.Y.); 182681814@st.usst.edu.cn (S.H.)

**Keywords:** nitrate reduction, electrocatalysis, hydrotalcite-like compounds, mechanism

## Abstract

In this research, nano Cu/Al–HTLCs, Co/Al–HTLCs and Cu/Co/Al–HTLCs were synthesized, characterized, and tested in electrolytic reduction nitrate. Experimental results showed that Cu/Al–HTLCs were less stable than Co/Al–HTLCs due to the Jahn–Teller effect. However, electrocatalytic activity of copper was superior to that of cobalt; thus, Cu/Co/Al–HTLCs were selected based on their stable crystalline structure and electrochemical activity. The optimized Cu_2_CoAl–HTLC was highly active in nitrate reduction, with two peaks for nitrate and nitrite reduction, respectively. Ammonia, nitrite and N-containing gases were found to be the final products of constant potential electrolysis at −0.54 and −0.74 V.

## 1. Introduction

Nitrate is a commonly detected form of inorganic nitrogen in municipal and industrial wastewater, surface water, groundwater, and drinking water. Nitrogen is an easily utilized nutritional element for the growth of algae, which can cause eutrophication as a result of algae blooming. In addition, nitrate can be reduced to nitrite in an anoxic environment, such as pickled or overnight food and intestinal tracts. Nitrite has an adverse effect involving oxidation of ferrous haemoglobin to its ferric form in the human body, which hinders transport of oxygen by haemoglobin. “Blue infant syndrome” is a typical adverse effect of nitrite-containing drinking water in infants and children [1]. A maximum limit of 0.73 mM nitrate is recommended by the World Health Organization for drinking water [2].

The frequently employed process for nitrate removal is biological denitrification in wastewater treatment plants, which requires suitable carbon source (volatile fatty acid preferred), pH, temperature, anoxic environment (dissolved oxygen < 0.5 mg/L), etc. [3]. Sometimes it is difficult to meet the critical conditions of biological denitrification for the treatment of industrial wastewater. For example, nuclear energy production, explosive and metal finishing industries usually discharge nitrate containing wastewater. Electrochemical methods are a promising way to remove the high concentrations of nitrate in the nuclear waste [2]. Another common question is the residue nitrate in secondary effluent of industrial wastewater treatment plant. It is reported that excess total nitrogen is a general reason for the underachievement of dyeing and finishing wastewater treatment. Those secondary effluents have been treated by biological processes, thus, it is more practical to improve their quality through chemical methods.

Electrochemical denitrification has attracted growing attention due to the advantages of easy automatic control, lack of carbon source requirements, absence of production of extra sludge, controlled reaction rate/selectivity, etc. Electrode materials have a direct relationship with the nitrate reduction rate as well as the final products. Copper is an effective cathode material with high electrochemical activity towards nitrate reduction and preferred selectivity to ammonia [4]. To improve electrode selectivity to nitrogen gas, various transition metals (such as Ni, Co, Zn, Pd, Fe, Sn, etc.) have been used to form copper alloy electrodes [4,5]. Generally, the diffusion and adsorption of nitrate on the surface of the cathode is the antecedent step of electrolytic reduction and, thus, limits the rate of reduction due to an electrolytic repulsive force between the cathode and the anions. It is necessary to investigate new electrode materials with high adsorption capacity and high electrocatalytic activity.

Hydrotalcite-like compounds (HTLCs) belong to anionic layered materials, and are sometimes called by their formal name of layered double hydroxides (LDHs). The general chemical composition of HTLCs can be expressed as follows: ${\left[M_{1-x}^{2+}M_{x}^{3+}{\left(\mathrm{OH}\right)}_{2}\right]}^{x+}{\left[{\left(A^{n-}\right)}_{x/n}\cdot {\mathrm{mH}}_{2}O\right]}^{x-}$, in which M^2+^ is a divalent metal cation (Mg^2+^, Co^2+^, Cu^2+^, Ni^2+^, Mn^2+^), M^3+^ is trivalent metal cation (Al^3+^, Fe^3+^, Co^3+^), A^n^-is interlayer exchangeable anion, and can be an inorganic anion (such as NO_3_^-^, SO_4_^2-^, CO_3_^2-^), organic anion, or metal coordination compound anion [6]. As reported by various researchers, crystalline HTLCs can be obtained with x ranging from a maximum value 0.50 to a less well-defined minimum value near 0.20 [7,8,9,10,11]. By changing the ratio of M^2+^ to M^3+^, different types of binary and ternary hydrotalcite-like substances can be prepared on the basis of maintaining the unique structure of hydrotalcite. The interlayer anions of HTLCs are exchangeable due to the hydrogen bonding between the laminate and interlayer anions. Generally speaking, the exchangeability of anions is related to their charge and properties. The order of exchangeability of common anions is NO_3_^−^, Cl^−^, F^−^, HPO_4_^2−^, SO_4_^2−^, and CO_3_^2−^ [12].

In the field of water pollution control, HTLCs are often used as adsorbents because of their large specific surface area and interlayer exchangeable ions. Generally speaking, the adsorption mechanism for neutral molecules and cations is mainly surface adsorption while, for anions, it is mainly through the interlayer ion exchange process. Compared with adsorption materials, such as activated carbon or zeolite, HTLCs have outstanding adsorption properties for anions in water treatment processes. For example, X-ray diffraction studies show that HTLCs can remove arsenic radicals in water by ion exchange, and the removal rate is as high as 97.6% [13]. Various types of HTLCs have been reported to be effective for the adsorption of nitrate from different solutions as well [14].

As an electrode material, HTLCs can provide a fast ion movement channel, which makes the ion release or absorption process involved in the electrode reaction easier. In the research of primary batteries, in order to overcome the disadvantage of the instability of α–Ni(OH)_2_, other metal cations are often introduced into the lattice to form layered composite metal hydroxides. For example, Al–substituted α–Ni(OH)_2_ by the co-precipitation method, which has high electrochemical activity [15]. The crystallinity, electrochemical stability and discharge capacity of the sample are directly related to the content of Al. Other researchers [16] synthesized Ni–Co–Al ternary layered metal hydroxides by the co-precipitation method, which exhibited the best capacitance performance when the nickel-cobalt ratio was 4:6.

Electrolytic reduction of anions is a challenge since the cathode usually repels the approaching of anions due to the electric force. The research hypothesis is that the adsorption of anions and small molecules on HTLCs can promote their reduction on the cathode electrochemically. To verify this hypothesis, copper-based HTLCs were synthesized and their electrochemical activity was investigated in the reduction of a representative anion, nitrate. Possible products were analysed and the relationship between the structure of HTLCs and nitrate reduction was discussed as well.

## 2. Materials and Methods

### 2.1. Materials and Reagents

Reagents, such as Cu(NO_3_)_2_3H_2_O, Co(NO_3_)_2_6H_2_O, Al(NO_3_)_3_9H_2_O, Na_2_CO_3_ and NaOH, were bought from Sinopharm Chemical Reagent Co. Ltd. (Shanghai, China), and used as received without further purification. Deionized water (Millipore Milli-Q system, resistivity ≥ 18.2 MΩ·cm) was used for the preparation of all solutions. Acetylene black, polyvinylidene fluoride (PVDF) and N–methylpyrrolidone (NMP) solution were purchased from KEJING Star Technology Co. Ltd. (Shenzhen, China). Nafion^®^ 117 membrane was obtained from Saosi Chemical Instrument Co., Ltd. (Hangzhou, China). Copper foil was bought from Amamda Hardware Trading Co., Ltd. (Shenzhen, China). After receipt, it was washed by ethanol for the removal of oil and grease, polished with 3000 mesh sandpaper and finally washed with distilled water. The pre-treated copper foil was dried in the shade for future use.

### 2.2. Preparation of HTLCs

The HTLCs were prepared by a modified co-precipitation method [10,17,18]. The experimental details were shown as follows. Solutions with 1.0 M total metallic ions with different molar ratios of Cu(NO_3_)_2_3H_2_O, Co(NO_3_)_2_6H_2_O and Al(NO_3_)_3_9H_2_O were prepared in distilled water and marked as solution A. Solution B was prepared using a mixture of 1.6 M NaOH and Na_2_CO_3_ with a molar concentration twice that of [Al^3+^]. Then, solutions A and B were added dropwise at a rate of 2.0 mL/min into 200 mL deionized water under vigorous stirring and 40 ± 3 °C. During the whole process, pH was maintained within the range from 9 to 10 by adding alkaline solutions (0.5 M Na_2_CO_3_ and 1.6 M NaOH). After that, the mixture was stirred for half an hour and aged at 65 ± 3 °C for 24 h. The precipitate was filtered, washed with deionized water to pH 7, dried, grounded and sieved by using a 100 screen mesh. The obtained powder is referred to as Cu/Al–HTLCs, Co/Al–HTLCs or Cu/Co/Al–HTLCs based on the composition of the solution A. The electrode was prepared by mixing HTLCs, acetylene black and PVDF at the ratio of 10:1:2; after thorough grounding, the reagents were mixed with NMP for 4 h. The mixture was used to coat pre-treated copper foil at a thickness of 150 μm; then, the electrode was dried in an 80 ± 3 °C vacuum oven for 6 h.

### 2.3. Characterization of HTLCs

Before use, the as-prepared Cu/Al–HTLCs, Co/Al–HTLCs and Cu/Co/Al–HTLCs were characterized by X-ray diffraction (XRD), field emission scanning electron microscopy (FE–SEM) and Fourier transform infrared spectroscopy (FT–IR), respectively. The crystal structures were scanned within the range of 5–80° by an X–ray diffractometer (XRD) (Bruker D8 Advance, Karlsruhe, Baden-Württemberg, Germany) with Cu-K_α_ radiation (40 kV, 40 mA). The molar ratios of metallic elements were quantified by inductively-coupled plasma optical emission spectrometer (ICP–OES) (Agilent 730, Palo Alto, CA, USA). The surface morphology was visualized by a field emission scanning electron microscope (Sigma500, Carl Zeiss AG, Oberkochen, Germany). The infrared spectra of the prepared HTLCs were obtained by a Fourier transform infrared spectroscope (FT–IR, Tensor27, BRUKER, Karlsruhe, Baden-Württemberg, Germany) within the wavenumber range from 4000 to 400 cm^−1^.

### 2.4. Electrochemical Measurement

Electrochemical analysis was performed on a CHI 660E electrochemical workstation (Chenhua Co. Ltd., Shanghai, China). Linear sweep voltammetry (LSV) was performed in a three-electrode cell with a Pt wire and Hg/HgO as the counter and reference electrodes, respectively. The working electrode was fixed 5 mm away from the counter electrode with a geometrical surface area of 0.5 cm^2^. The 8 mL electrolyte consisted of 1.0 M NaOH and 0.01–0.2 M nitrate solution. The scans were performed at the rates of 10–100 mV/s within the potential range from −0.2 to −1.4 V.

H-type electrolysis cell separated by a Nafion^®^ 117 membrane was used for the constant potential electrolysis. In the cathodic compartment, 45 mL mixture of 1.0 M NaOH and (95 ± 6) mg N/L NaNO_3_ was used as the electrolyte. Working electrode with a geometric surface area of 8 cm^2^ was placed inside the compartment. In the anodic compartment, Pt electrode (2 × 4 cm) was immersed into a NaOH solution. The potentials of −0.54 and −0.74 V were applied separately based on the LSV results. Each time, 0.5 mL sample was taken and diluted for 20 times, and then measured immediately to avoid possible changes in nitrogen content.

### 2.5. Analytical Methods

Nitrate concentration was measured through a spectrophotometric method at 220 and 275 nm by using a UV–VIS spectrophotometer (Shimadzu UV-2600, Tokyo, Japan) [19]. The influence of nitrite was eliminated by adding 0.8% aminosulfonic acid solution. Ammonia was determined through the Nessler’s method [20]. The aqueous nitrite concentration was analysed by N-(1-naphthyl)-ethylenediamine spectrophotometric method [21]. Total nitrogen was monitored through a TOC/TN analyser (Multi N/C 3100, Analytic Jena Company, Jena, Germany). The pH was measured by a pH meter (PHS-2 C; Leici Company, Shanghai, China).

## 3. Results and Discussion

### 3.1. Characterization of The Prepared HTLCs

**Figure 1:**
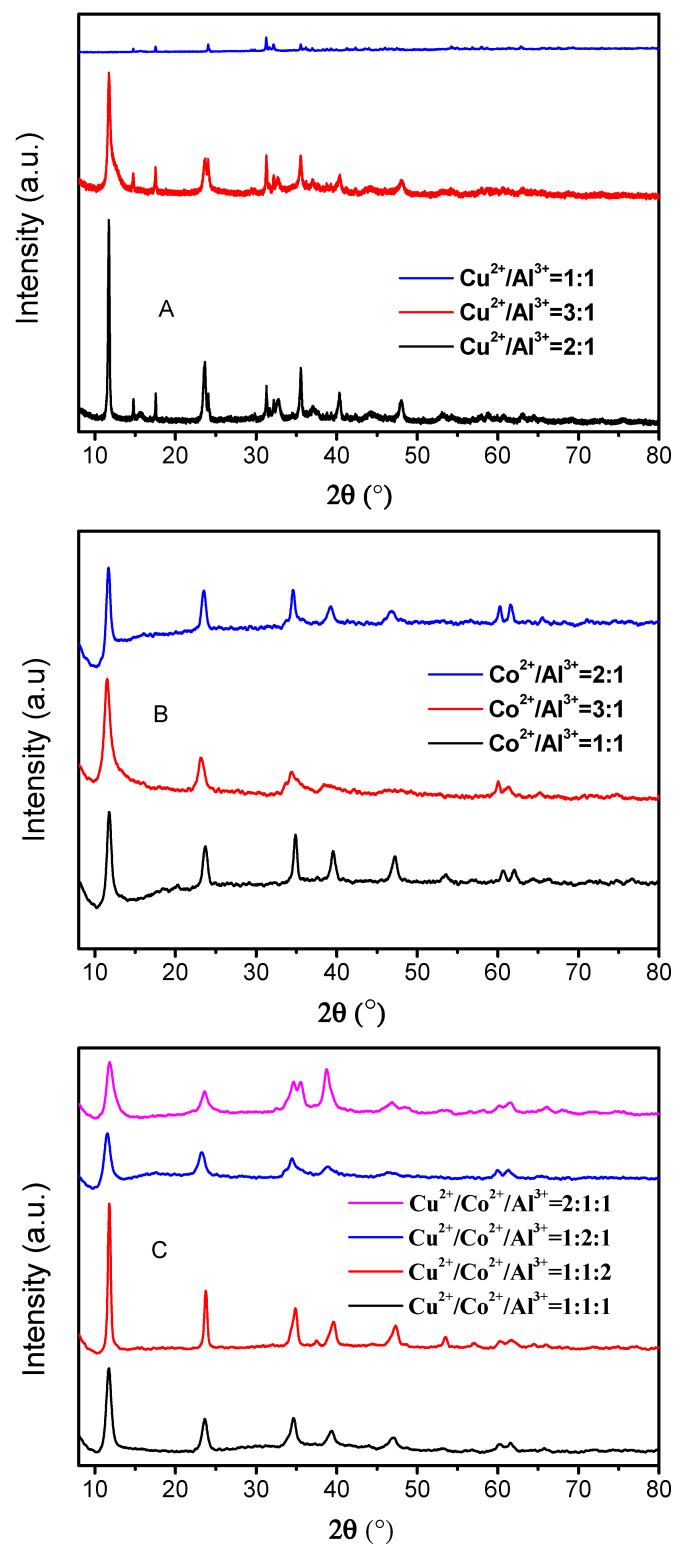
XRD patterns of the prepared HTLCs: (**A**) Cu/Al–HTLCs; (**B**) Co/Al–HTLCs; (**C**) Cu/Co/Al–HTLCs.

The XRD patterns of Cu/Al–HTLCs (1:1, 2:1 and 3:1), Co/Al–HTLCs (1:1, 2:1 and 3:1), and Cu/Co/Al–HTLCs (1:1:1, 1:1:2, 1:2:1 and 2:1:1) are shown in Figure 1A–C, respectively. In Figure 1A, the high intensity diffraction peaks at 2θ of 11.7°, 24.1°, 35.6°, 40.3° and 48.0° can be assigned to the (003), (006), (009), (015) and (018) diffraction planes of Cu/Al–HTLCs, respectively [22]. The molar ratio of 2:1 resulted in a better crystalline feature compared to that in the case of the 1:1 and 3:1 ratios. The presence of several tiny peaks might be ascribed to the generation of other co-precipitates such as Cu(OH)_2_ or Al(OH)_3_ [23]. It was assumed that excessive amount of Cu^2+^ can form an octahedral structure with hydroxyl groups, which hindered the generation of Cu/Al–HTLC [23]. In Figure 1B, diffraction peaks were detected at 2θ of 11.7°, 23.9°, 34.6°, 40.2°, 46.7°, 61.4° and 61.6°, which are typically indexed to the (003), (006), (009), (015), (018), (110) and (113) diffraction planes of Co/Al–HTLCs, respectively [24]. Significant symmetrical peaks were observed at various Co:Al ratios indicating the formation of flawless crystals under various conditions. Copper is an excellent element for electrolytic reduction process; however, in case of pure CuAl–HTLCs, the formation of copper compound is energetically preferred to that of HTLCs, and the crystal structure of HTLCs can be affected [23]. Thus cobalt was introduced to produce a crystal lattice of Cu/Al–HTLCs as shown in Figure 1C; main diffraction peaks were detected at 2θ of 11.6°, 23.7°, 34.7°, 39.3°, 47.1° and 61.3°, which are ascribed to (003), (006), (009), (015), (018) and (110) diffraction planes of Cu/Co/Al–HTLCs, respectively. The Cu:Co:Al molar ratio of 1:1:2 produced sharp and symmetrical peaks indicating the formation of a better crystalline. Calculations performed according to the Bragg’s law at 2θ = 12° determined the distance between different layers as 0.7498, 0.7528, 0.7698 and 0.7583 nm for the Cu:Co:Al ratios of 1:1:2, 1:1:1, 1:2:1 and 2:1:1, respectively. All distances were slightly smaller than that in the typical Mg/Al–HTLCs (0.7837 nm). Generally speaking, the interlayer distance of HTLCs was affected mainly by the type of interlayer anions, such as CO_3_^2−^, Cl^−^, NO_3_^−^, etc. However, larger radius of Cu^2+^ and Co^2+^ on the laminate can occupy more interlayer space than that of Mg^2+^, which led to a smaller interlayer distance. As shown in Table 1, the molar ratios of metallic ions measured by ICP–OES were close to those of the theoretical values, which again proved that the HTLCs were synthesized successfully.

**Figure 2:**
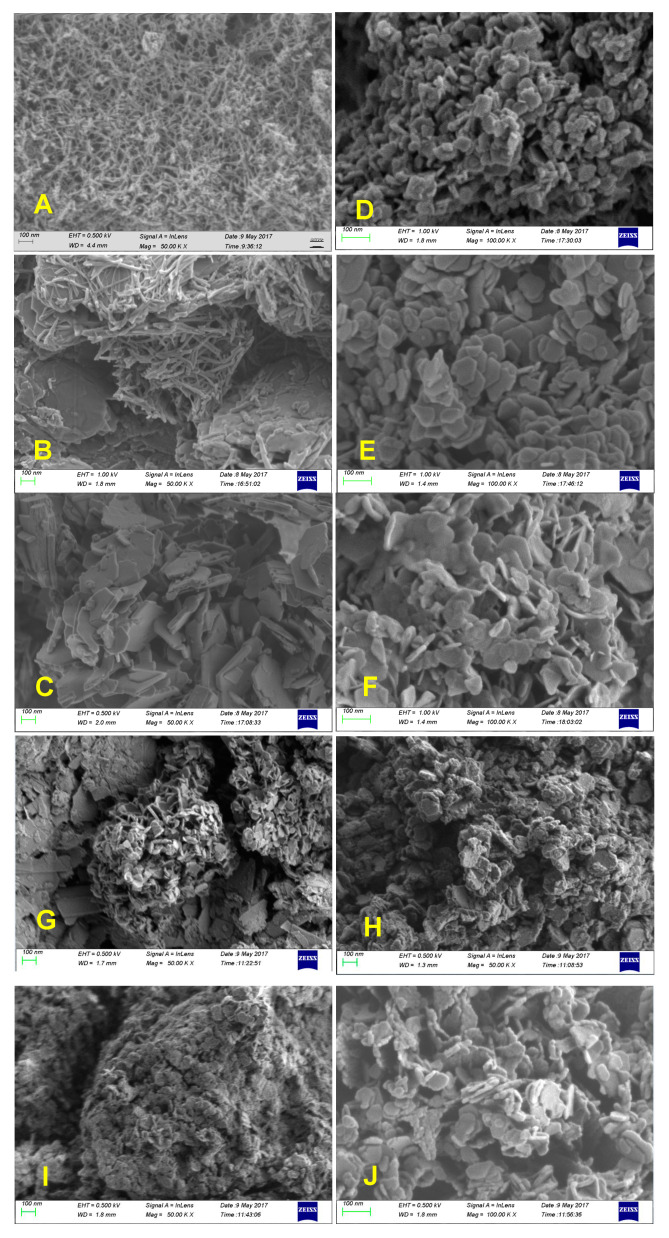
FE–SEM photographs of the prepared HTLCs: (**A**) 1:1; (**B**) 2:1; (**C**) 3:1 Cu/Al–HTLCs; (**D**) 1:1; (**E**) 2:1; (**F**) 3:1 Co/Al–HTLCs; (**G**) 1:1:1; (**H**) 1:1:2; (**I**) 1:2:1; (**J**) 2:1:1 Cu/Co/Al–HTLCs.

The FE–SEM photographs of Cu/Al–HTLCs, Co/Al–HTLCs and Cu/Co/Al–HTLCs with various components are shown in Figure 2A–J, respectively. Comparison of Figure 2A with Figure 2B,C indicates that a better layered structure was observed at the Cu:Al molar ratios of 2:1 and 3:1, respectively; this is in agreement with the result of the XRD diffraction patterns. Low copper content resulted in a collapse of the typical layered structure due to the Jahn–Teller effect. It was assumed that Cu^2+^ occupied the symmetric point of the octahedron in the LDH laminate. Thus in general, the crystal cells of HTLCs are still symmetrical on the whole. When n(Cu^2+^): n(Al^3+^) equals to 1:1, Cu^2+^ was not enough to occupy all the symmetric sites in the unit cell of LDH. In the case of Co/Al–HTLCs, the molar ratio of Co:Al had little effect on the stable layered structure as shown in Figure 2D–F. Unlike copper ion, cobalt ion may have a low affinity to the hydroxyl group. Furthermore, the introduction of cobalt ion to the crystal lattice of Cu/Al–HTLCs can reduce the effect of copper ion and form relatively stable layered structure as shown in Figure 2G–J similar to the results of the XRD diffraction patterns. The dimensions of Cu/Co/Al–LDHs were mostly within 100 nm with a layer thickness less than 10 nm, which can be formed by an overlay of several laminates. When the cobalt content was increased, the dimensions of the lamellar and interlayer spacing were significantly reduced. When the copper content was increased, the dimensions of the lamella and interlayer spacing were significantly increased in the best sample in Figure 2J (Cu:Co:Al = 2:1:1), which may be beneficial for the adsorption and catalysis process.

**Figure 3:**
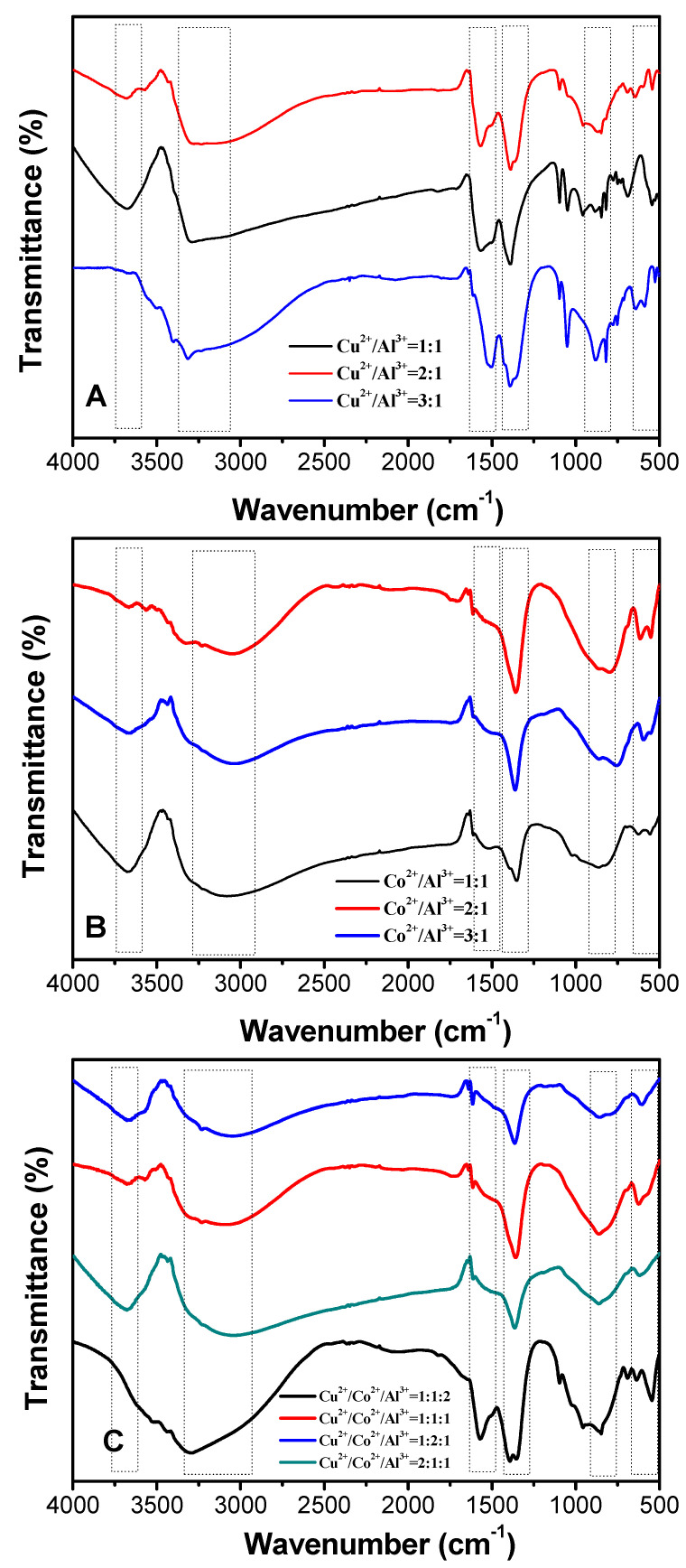
FT–IR spectra of the prepared HTLCs: (**A**) Cu/Al–HTLCs; (**B**) Co/Al–HTLCs; (**C**) Cu/Co/Al–HTLCs.

To verify the possible functional groups, FT–IR analysis was performed for HTLCs of various compositions. In the case of Cu/Al–HTLCs, typical FT–IR spectra were recorded for the Cu:Al molar ratios of 1:1, 2:1 and 3:1 as shown in Figure 3A. The band near 3430 cm^−1^ was assigned to the stretching mode of the hydroxyl group. Compared with a typical band (3600 cm^−1^) of a free hydroxyl group, the band moved towards a lower wavenumber, which can be interpreted as the formation of a hydrogen bond between interlayer H_2_O or CO_3_^2−^ and a hydroxyl group in the laminate [25,26]. The band near 500 cm^−1^ was attributed to the M–O modes (M: Cu or Al); the band near 1350 cm^−1^ was assigned to the ν3 antisymmetric stretching vibration of carbonate in the interlayers; the weak band observed at 1620 cm^−1^ was assigned as the –OH bending vibration from the interlayer water [12,27]. Figure 3B shows the FT–IR spectra of Co/Al–HTLCs at the Co:Al molar ratios of 1:1, 2:1 and 3:1. The stretching of the hydroxyl groups in the laminate were observed as a band near 3210 cm^−1^. The weak band at 1630 cm^−1^ belonged to the interlayer water; the band at 1358 cm^−1^ can be ascribed to the ν3 antisymmetric stretching vibration of carbonate; the bands near 550 cm^−1^ are commonly recognized as the M–O (M: Co or Al) stretching peaks. Figure 3C shows the FT–IR spectra of Cu/Co/Al–HTLCs at the Cu:Co:Al molar ratios of 1:1:2, 1:1:1, 1:2:1 and 2:1:1. Similar bands at 3250–3350 cm^−1^, 1600 cm^−1^, 1340–1400 cm^−1^, 880 cm^−1^ and 550 cm^−1^ can be attributed to the stretching vibration peak of –OH in the laminate, bending vibration peak of the O–H bond in interlayer H_2_O, ν3 antisymmetric stretching vibration of carbonate, ν2 vibration peak of CO_3_^2−^, and M–O (M: Cu, Co or Al) stretching peaks, respectively.

### 3.2. Electrocatalytic Performance

**Figure 4:**
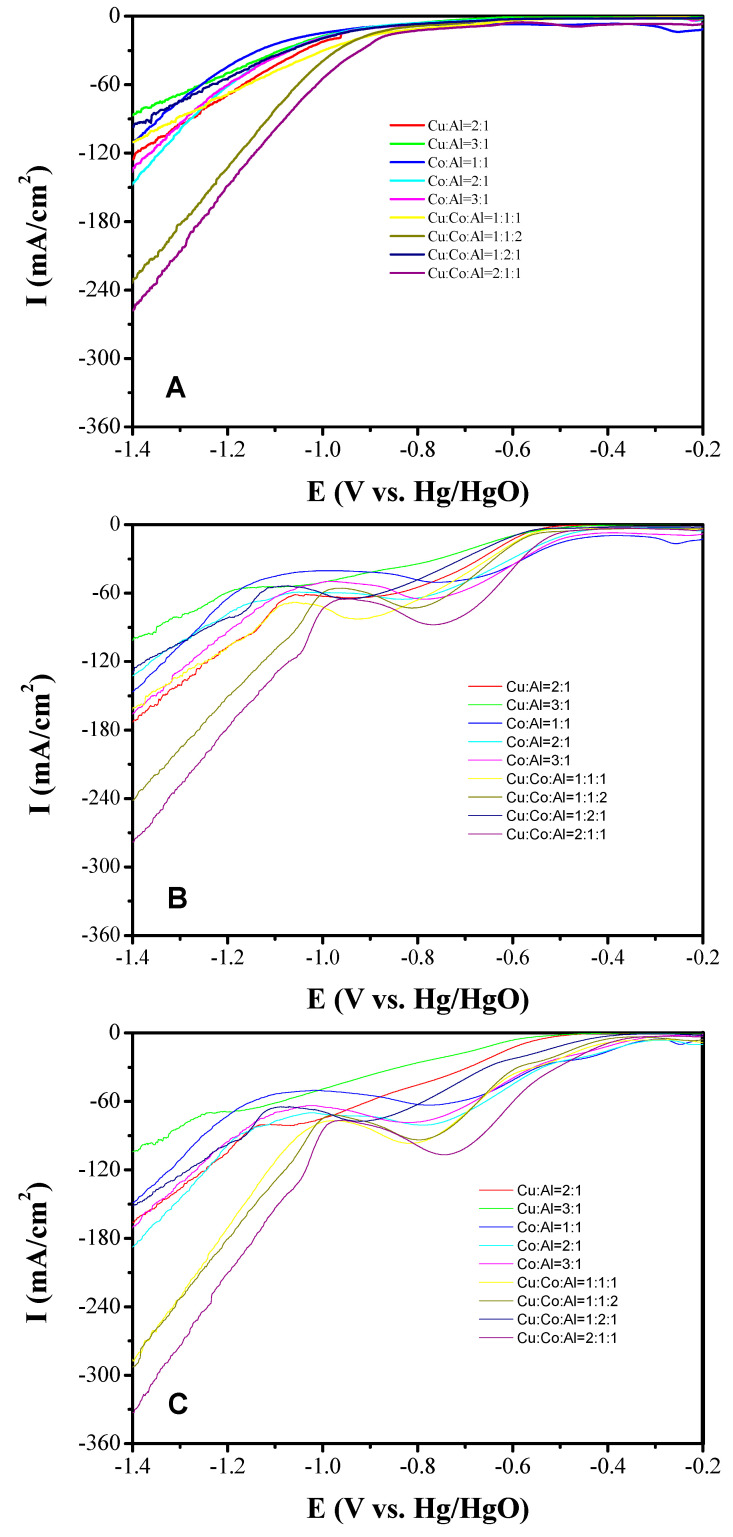
Linear sweep voltammograms on various electrodes: (**A**) 1 M NaOH; (**B**) 1 M NaOH + 0.1 M NaNO_2_; (**C**) 1 M NaOH + 0.1 M NaNO_3._

To investigate the electrocatalytic activity of various HTLCs electrodes, linear sweep voltammograms (LSV) were recorded in 1 M NaOH, 1 M NaOH + 0.1 M NaNO_2_ and 1 M NaOH + 0.1 M NaNO_3_. Figure 4A shows the electrolytic activities of various electrodes in 1 M NaOH solution. The onset potential for the current density of 10 mA/cm^2^ was within the range from −0.72 V to −0.94 V (vs. Hg/HgO) for various HTLCs, which can be calibrated to a normal hydrogen electrode (NHE) with a value from −0.622 V to −0.842 V [28,29]. The reducing peak may be ascribed to two main electrolytic processes. One process involves the hydrogen evolution (HER) with a standard potential of −0.828 V in alkaline solutions and another process may be related to reduction of copper or cobalt hydroxides with standard potentials of −0.222 V and −0.730 V, respectively.

Reyter et al. pointed out that the HER usually starts with a potential of −1.4 V on the surface of a pure copper electrode [30]. The higher reduction potential of HTLC electrodes indicates that HER is substantially easier, which may be explained by the adsorption of the H_2_O molecules into the interlayer of HTLCs. HTLC-related material may have a bright future for the electrolytic, or even photocatalytic, splitting of water processes. It should be noted that Cu/Co/Al–HTLCs with the Cu:Co:Al molar ratio of 2:1:1 showed a significantly higher catalytic activity for HER compared with that of the other HTLCs. Multiple metal ions may have a synergistic effects in electrolytic reduction, which requires additional studies of the HER process on the surface of various HTLCs electrodes.

Figure 4B shows electrolytic reduction of nitrite by various HTLCs in 1 M NaOH + 0.1 M NaNO_2_ solution. Compared with Figure 4A, another significant reduction peak is detected with a starting potential near −0.5 V, which is commonly assigned to the reduction of nitrite to ammonia [31,32]. The peak intensity was higher than that of the pure copper electrode as shown in the previous studies indicating lower activation energy of the reaction [33,34]. Cu/Co/Al–HTLCs with a molar ratio of 2:1:1 showing the highest reduction current density of 88 mA/cm^2^ compared to that of all other HTLCs. Moreover, the highest peak potential of −0.766 V was higher than that in other HTLCs implying lower activation energy of nitrite reduction. This phenomenon can be explained by the synergistic effect of bimetallic atoms in Cu/Co/Al–HTLCs since typically copper has a high affinity to O, while cobalt may have a high affinity to N in NO_2_^-^ ion [35,36].

The electrolytic reduction of nitrate by various HTLCs in 1 M NaOH + 0.1 M NaNO_3_ solution is shown in Figure 4C. Compared with Figure 4B, a new reduction peak is observed with a starting potential of −0.3 V representing the reduction of nitrate. This starting potential shifted towards positive position compared with copper electrode described in the previous reports [37]. A possible product was suggested to be nitrite or hydroxylamine on the copper electrode. Co/Al–HTLC with a Co:Al molar ratio of 1:1 showed the highest electrolytic activity of all the HTLCs in nitrate reduction with a peak potential near −0.45 V and a peak current density of 22 mA/cm^2^. However, in the subsequent reduction of nitrite to ammonia, Cu/Co/Al–HTLCs showed the highest electrolytic activity with a peak potential near −0.75 V and a peak current density of 107 mA/cm^2^, which was superior to that of the pure copper electrode described in the previous reports [32]. 

**Figure 5:**
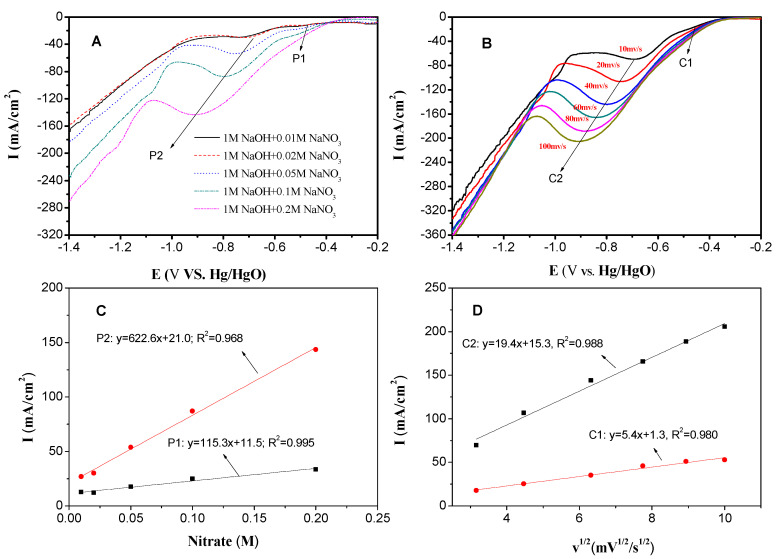
LSV curves of Cu_2_CoAl-HTLC in alkaline solution under various: nitrate concentrations (**A**) & scan rates (**B**); Linear relationship between peak currents and nitrate concentrations (**C**) & root of scan rates (**D**).

In summary, Cu/Co/Al–HTLC with the Cu:Co:Al molar ratio of 2:1:1 showed a good crystalline structure and excellent electrocatalytic capacity; thus, it was selected for the subsequent experiments and is referred to as Cu_2_CoAl–HTLC [26,38]. In Figure 5, different scan rates and initial nitrate concentrations were employed to further investigate the electrolytic activity of Cu_2_CoAl–HTLC. As shown in Figure 5A, two reduction peaks (P1 and P2) were found for the reduction of nitrate and nitrite, respectively. As the initial nitrate concentration increased from 0.01 to 0.2 M, the peak potential for P1 moved from −0.50 V to −0.55 V, and the peak current increased from 12 mA/cm^2^ to 43 mA/cm^2^ correspondingly. Simultaneously, the P2 shifted from −0.72 V to −0.92 V, together with a rise of the peak current from 30 mA/cm^2^ to 144 mA/cm^2^. As shown in Figure 5C, the peak current density had a linear relationship with initial nitrate concentration (R^2^: 0.968–0.995) [24,36]. The peak shifted towards negative potential with nitrate concentration, indicating that the reduction of nitrate was an irreversible process [33].

Figure 5B showed LSV of nitrate reduction in 1 M NaOH + 0.1 M NaNO_3_ solution with scan rates 10–100 mV/s. Similarly, two reduction peaks (C1 and C2) were observed around −0.47 to −0.54 V and −0.69 to −0.90 V, respectively. Both peak potentials for C1 and C2 shifted towards negative, which suggested irreversible reactions as well. Furthermore, when scan rates increased from 10 to 100 mV/s, the peaks currents for C1 and C2 increased from 18 and 70 mA/cm^2^ to 56 and 206 mA/cm^2^. As shown in Figure 5D, the peak current density also had a linear relationship with the root of scan rates. Since the regression results showed that it did not pass the original point, which indicated that nitrate was pre-adsorbed on the surface of Cu_2_CoAl–HTLCs.

**Figure 6:**
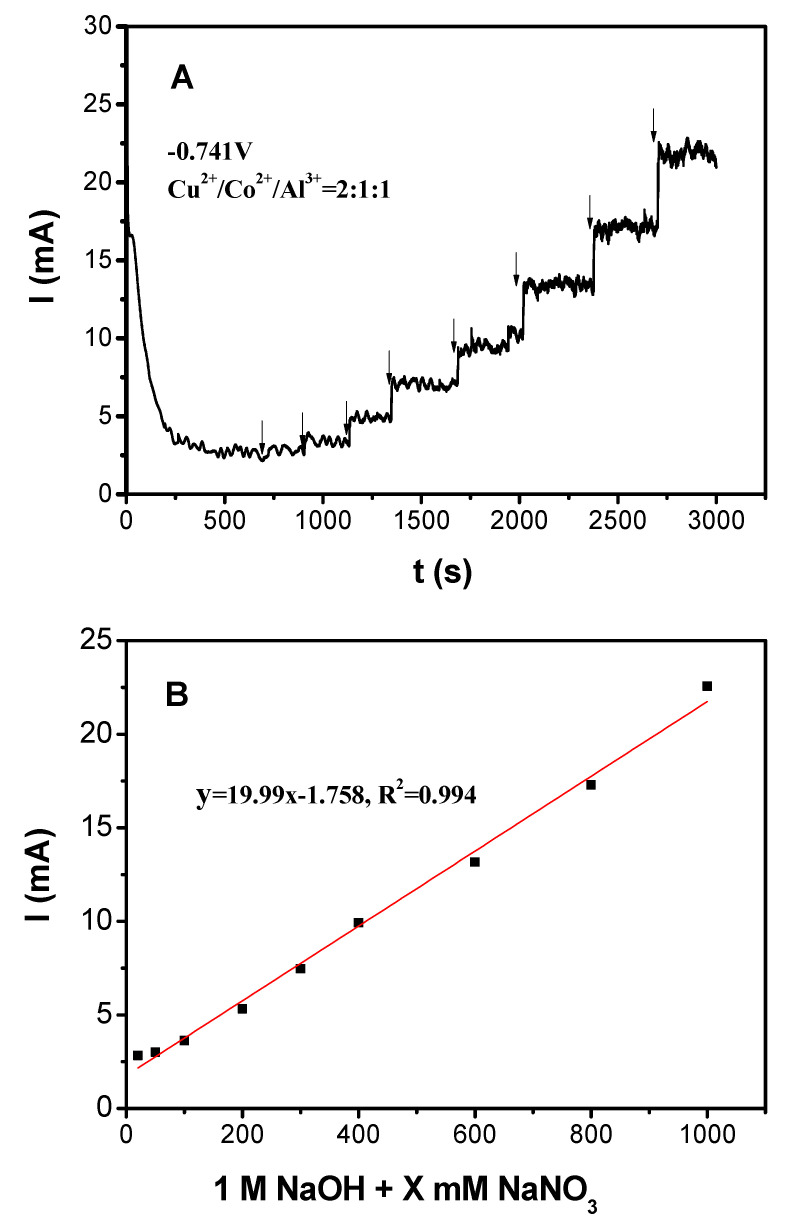
(**A**) Amperometric response of the Cu_2_CoAl–HTLC at an applied potential of −0.741V (vs. Hg/HgO) to the successive addition of nitrate; (**B**) linear relationship between nitrate concentration and the corresponding current.

Figure 6A illustrates a typical amperometric response of the Cu_2_CoAl–HTLC at an applied potential of −0.741V (vs. Hg/HgO) to the successive addition of nitrate. The electrolyte employed in the cathodic region was 1 M NaOH to minimize the effect of hydrogen evolution reaction [26,39,40]. In the beginning, a steady background noise was observed around 500 s. With the addition of nitrate, a rapid and sensitive current response was observed with the as-prepared working electrode [36]. Moreover, the corresponding current signal was found to change linearly with aqueous nitrate concentration within the experimental range as shown in Figure 6B. This phenomenon indicated that Cu_2_CoAl–HTLC had the potential for the quick analysis of nitrate in the aqueous phase [29].

**Figure 7:**
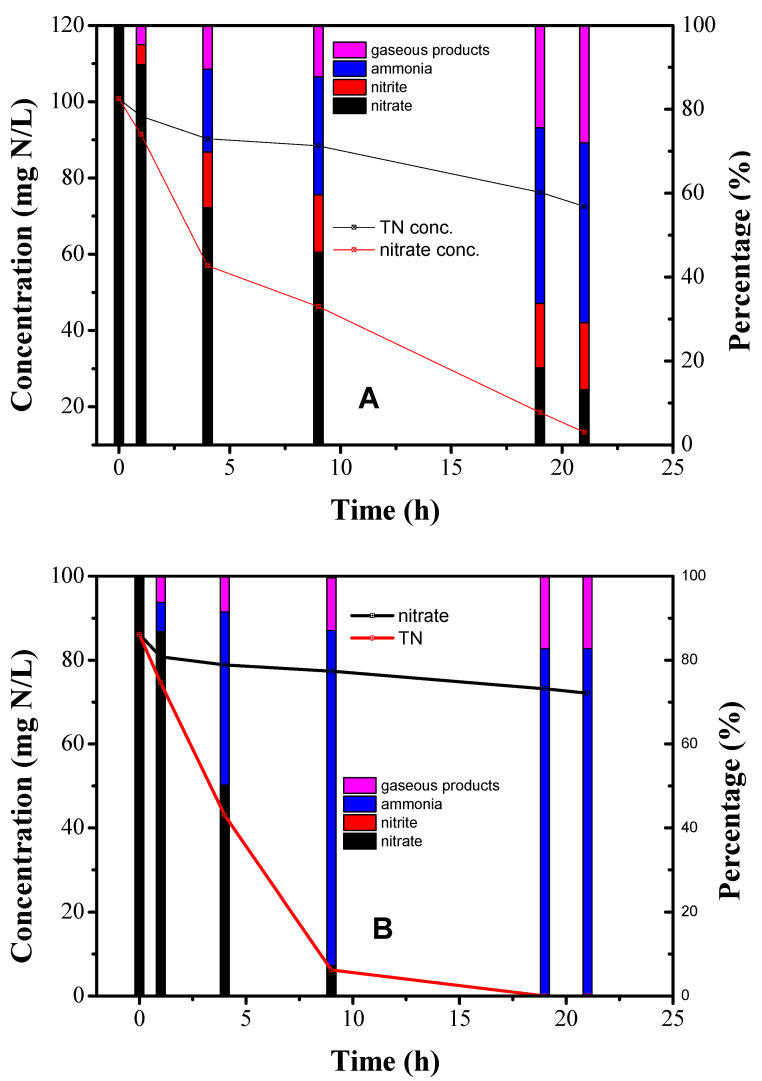
The transformation of nitrogen during constant potential electrolysis at: (**A**) −0.54 V; (**B**) −0.74 V.

To verify the possible products, constant potential electrolysis was performed at −0.54 V and −0.74 V. The data of Figure 7A indicate that nitrate and total nitrogen (TN) decreased significantly during electrolysis at −0.54 V in a time-dependent manner. The removal of nitrate followed the apparent first-order kinetics and the kinetic constant was determined to be 0.09 h^−1^. A reduction in TN can be attributed to the generation of N-containing gases [35]. Ammonia, nitrite and gaseous products contributed 43%, 16% and 28% of the final products, respectively. Figure 7B shows the results of constant potential electrolysis at −0.74 V. Nitrate removal followed apparent first-order kinetics with a kinetic constant of 0.30 h^−1^, which was 3.3 times faster than that at −0.54 V. Lower negative potential was beneficial for nitrate removal and generally consumed more energy. The final products consisted of 83% ammonia and 17% N-containing gases. Comparison with the results in Figure 7A, it indicates that ammonia was preferred as a final product at a negative potential.

From the point of TN removal, the generated ammonia can be further removed by indirect oxidation at the anode [40]. In this situation, the chosen anode should be beneficial for the evolution of chlorine. IrO_2_ or RuO_2_ anode might be a better choice than Pt anode, since they had a lower chlorine evolution over potential. Our previous research proved that 86% nitrate could be converted to gaseous nitrogen with the addition of 300 mg/L chloride ion in one electrolytic cell by using RuO_2_/Ti and Cu as the electrodes [32]. Considering the reactor design, a plug flow system without membrane separation is suggested with mesh or foamed electrodes. The influent containing certain amount of nitrate firstly passes the cathodic region with the transformation to ammonia, followed by the anodic area with the transformation of ammonia to gaseous products.

## 4. Conclusions

For the synthesis of HTLCs, cobalt was superior than copper in the formation of a stable layered structure; however, copper was beneficial for the electrolytic reduction of nitrate. Thus, optimized hydrotalcite Cu/Co/Al–HTLCs were formed with both cobalt and copper. Cu_2_CoAl–HTLC with the molar ratio of 2:1:1 showed the best electrochemical activity in nitrate reduction. The phenomenon of pre-adsorption of nitrate was observed, which promoted the nitrate reduction. For the electrolysis at −0.54 V, ammonia, nitrite and N-containing gases contributed 43%, 16% and 28% of the final products. However, at −0.74 V, 83% ammonia and 17% N-containing gases were determined in the final products. Negative potential was beneficial for nitrate reduction but produced more ammonia.

## Figures and Tables

**Table 1:** The molar ratio of various metallic components measured by ICP–OES.

Sample	CuAl–LDH			CoAl–LDH			CuCoAl–LDH			
	n(Cu^2+^): n(Al^3+^):			n(Co^2+^): n(Al^3+^)			n(Cu2^+^): n(Co2^+^): n(Al3^+^)			
Theoretical molar ratio	1:1	2:1	3:1	1:1	2:1	3:1	1:1:1	2:1:1	1:2:1	1:1:2
Measured molar ratio	1.1:1	2.1:1	3.1:1	1.2:1	2.1:1	3.3:1	1:1.1:1.1	1.9:1:1.1	1:2.2:1.3	1:1.1:2.0

## Impact Statement

To alleviate the electrostatic repulsion of anions on the cathode, a novel and highly effective electrode material, Cu_2_CoAl–HTLC, has been synthesized for the adsorption and electrochemical reduction of nitrate.
